# Monoamine oxidase B is associated with γ-secretase in cultured neurons and in brain

**DOI:** 10.1186/1750-1326-8-S1-P69

**Published:** 2013-10-04

**Authors:** Sophia Schedin Weiss, Birgitta Wiehager, Bengt Winblad, Susanne Frykman, Lars Tjernberg

**Affiliations:** 1 NVS/Karolinska Institutet, Stockhom, Sweden

## Background

Monoamine oxidase B (MAOB) is a mitochondrial outer membrane protein that degrades monoamines such as dopamine. Thus, MAOB inhibitors are used to treat for instance Parkinson’s disease patients. MAOB inhibitors, however, have neuroprotective properties that are independent on the monoamine-degrading capabilities and it is possible that MAOB have additional unrevealed functions.

In an affinity pulldown/mass spectrometric screen for potential regulators of APP processing, we found MAOB to be associated with γ-secretase in membranes prepared from brain, and data from co-immunoprecipitation experiments supported this finding. siRNA experiments showed that reduced levels of MAOB resulted in reduced Aβ production, with only minor effects on the release of Notch intracellular domain (manuscript in preparation).

In the present study, we use the proximity ligation assay (PLA), which gives a fluorescent signal when two target proteins are less than 40 nm apart, to study the MAOB-γ-secretase interaction in primary neurons and brain.

## Materials and methods

PLA was performed on fixed and permeabilized mouse primary hippocampal neurons and deparaffinized thin sections from human brain cortex. The protocol from Olink Bioscience was essentially followed. Validated rabbit anti-MAOB and mouse anti-PS1 loop antibodies were used in the first incubation step, followed by secondary antibodies conjugated to oligonucleotide strands (MINUS and PLUS) in the second step. Reagents were from Olink Bioscience, and images were acquired using a Zeiss 510 confocal microscope (40x oil immersion objective). For immunohistochemistry, thin sections (7 µm) of formaldehyde fixed, paraffin embedded human frontal cortex were de-waxed and rehydrated. The sections were heated to 120°C for efficient epitope retrieval and stained for MAOB which was visualized using the DAB/H_2_O_2_ method. Micrographs were acquired with a brightfield microscope.

## Results

A clear interaction between γ-secretase and MAOB was observed in the primary neurons. The neuronal interaction was also supported by PLA studies on human brain sections. Immunohistochemistry using two different MAOB antibodies showed intense staining of neurons in human brain frontal cortex and hippocampus. The neuronal staining was more intense in AD than in control brains. Altogether, these data support that the interaction between MAOB and γ-secretase in neurons may be an interesting target for AD treatment.

## Conclusions

By several independent techniques, we have shown that monoamine oxidase B is associated to γ-secretase in neurons as well as in brain. Interestingly, siRNA mediated gene silencing of MAOB in cells reduces Aβ production, but not Notch processing. Thus, we conclude that interfering with the γ-secretase-MAOB interaction could be a therapeutic strategy for lowering Aβ levels in human brain.

